# State of Health for Lithium-Ion Batteries Based on Explainable Feature Fragments via Graph Attention Network and Bi-Directional Gated Recurrent Unit

**DOI:** 10.3390/s25195953

**Published:** 2025-09-24

**Authors:** Wenpeng Luan, Hanju Cai, Xiaohui Wang, Bochao Zhao

**Affiliations:** School of Electrical and Information Engineering, Tianjin University, Tianjin 300072, China

**Keywords:** lithium-ion battery, state of health estimation, incremental capacity analysis, graph attention network, bi-directional gated recurrent unit

## Abstract

Accurate lithium-ion battery state of health estimation is critical for safety and range anxiety mitigation. Existing methods often lack interpretability in the extraction of feature fragments and fail to model spatial correlations between features. To address these gaps, this paper introduces a novel framework centered on interpretable feature engineering and synergistic spatial–temporal learning. The core novelty lies in using incremental capacity (IC) analysis on charging data, captured by onboard sensors, to dynamically select a 0.1 V voltage window based on IC peaks, ensuring the extracted voltage and capacity fragments are physically meaningful. These fragments are then transformed into graph-structured data, enabling a graph attention network and a bi-directional gated recurrent unit to effectively capture both spatial dependencies and temporal degradation trends, with a residual connection optimizing the network. Validation on two public benchmark datasets demonstrates the model’s superiority, achieving an average mean absolute error of 0.561% and a root mean square error of 0.783%. Furthermore, the model exhibits a low computational footprint, requiring only 1.68 MFLOPs per inference, and its fast inference time of 17.55 ms on an embedded platform confirms its feasibility for practical deployment.

## 1. Introduction

In the context of global climate governance and energy decarbonization transition, the green change in transportation has become a key initiative to achieve “dual-carbon” goal [1]. Leveraging significant technical and economic advantages such as high energy density, long cycle life, and a wide operating temperature range, lithium-ion batteries have emerged as the core driving force for the development of electric vehicles (EVs) [2]. However, with their widespread adoption in EVs, safety hazards arising from performance degradation have become increasingly prominent, representing a key bottleneck hindering industry progress. Consequently, developing high-precision and practical state of health (SOH) estimation algorithms stands as a key technological challenge for ensuring safe and reliable operation of EVs and promoting sustainable development of the industry [3]. The performance of these algorithms is critically dependent on the quality of data from onboard sensors, such as voltage and current sensors within the battery management system (BMS), making sensor data reliability a decisive factor for accurate and robust estimation.

Extensive research has been conducted on battery SOH assessment, with approaches that can be summarized as direct measurement methods, model-driven methods and data-driven methods [4,5]. Direct measurement methods determine SOH by measuring capacity or internal resistance, including ampere-hour counting method and electrochemical impedance spectroscopy. While these techniques are simple and easy to implement, they must be carried out in specific environments and are difficult to apply online [6]. Model-driven methods enable SOH estimation by describing and characterizing the internal electrochemical mechanisms or external behavioral features of the battery [7]. They mainly include electrochemical models and equivalent circuit models [8]. Model-based methods are interpretable and logical, enabling the estimation of battery capacity from the intrinsic causes of aging. However, such methods require periodic updating of parameters and have limited generalization capabilities, making them difficult to apply to different battery types and operating conditions.

In recent years, the integration of artificial intelligence and machine learning has become an indispensable approach for addressing the complex challenges of battery reliability and health management [9,10]. A comprehensive review by Bello et al. [11] provides a holistic analysis of this evolution, meticulously detailing various battery degradation mechanisms and highlighting the critical role of machine learning in enabling advanced prognostics and health management. Their work particularly emphasizes the application of intelligent algorithms for SOH estimation. These data-driven methods do not require an understanding of the complex internal mechanisms of the battery, instead, they utilize historical data extracted from the charging and discharging processes or adopt an end-to-end approach to achieve SOH estimation [12]. Typically, traditional machine learning algorithms, such as support vector machines [13,14], Gaussian process regression [15,16,17,18], and random forests [19,20,21], implement SOH estimation by constructing models and using manually extracted features and correlation analysis. However, these methods rely on manually extracted health indicators and are insensitive to data variations, resulting in limited generalization [22]. In contrast, deep learning methods such as long short-term memory network (LSTM) and gated recurrent unit (GRU) can automatically extract features and perform end-to-end learning, avoiding complexity of manual feature extraction and significantly improving accuracy of SOH estimation [23]. Recent breakthroughs are being driven by the adoption of more sophisticated architectures. For instance, temporal convolutional networks, often enhanced with optimization algorithms or attention mechanisms, prove effective in capturing complex temporal patterns within battery operational data [24,25]. These approaches demonstrate the power of advanced neural networks in modeling the long-term dependencies inherent in battery signals. Although data-driven approaches show great potential in the field of battery condition estimation, their inherent “black-box” nature poses significant limitations. This opacity means that key features extracted from complex models often lack intuitive physical meaning, making it difficult to effectively integrate chemical and physical degradation processes into the estimation model. Incremental capacity (IC) analysis, as an important tool for extracting features, converts voltage plateau of charging curve into a differential capacity curve with significant peaks, enabling a coupled analysis of the battery’s health degradation process and its internal electrochemical mechanisms [26]. Li et al. [27] proposed an improved IC curve acquisition method based on reference voltage, from which characteristic variables were extracted, and SOH estimation was realized using gaussian process regression, which verified that the proposed method could provide accurate SOH estimation. Xu et al. [28] implemented SOH estimation based on peak features of IC curves using Transformer model with multi-head attention mechanism and verified that the proposed method provided higher accuracy than machine learning methods.

In real-world scenarios, batteries are often charged and discharged from various states of charge, making it difficult to obtain complete charging and discharging curves [29]. This uncertainty directly affects reliability of SOH estimation methods based on feature extraction [30,31]. Therefore, the complexity and stochasticity of actual charging and discharging process must be taken into account when developing SOH estimation methods. To address this, Gao et al. [32] proposed a new method for SOH estimation based on a novel state-space model using IC curves which utilized partial charging data. Lin et al. [26] proposed an advanced integrated learning framework technique to estimate SOH using arbitrary charging voltage information.

In addition, most existing estimation methods consider only the temporal information of sequences while ignoring their spatial information. In recent years, the graph neural network (GNN), which can effectively process spatial data and extract spatial features, has attracted attention of scholars in field of battery SOH estimation [33]. Yao et al. [34] first used GNN for battery capacity prediction problem, but it requires complete charging data to extract features, which is not suitable for practical applications. He et al. [35] proposed an approach based on graph convolution and LSTM to realize SOH estimation for batteries with multi-stage constant current–constant voltage fast charging strategy. Xu et al. [36] proposed a feature extractor that combines a residual (Res) structure with an attention mechanism. This extractor is designed to automatically capture temporal and spatial information from aging features. These features were then used to estimate battery capacity with an LSTM model enhanced by a graph attention network (GAT). However, a significant drawback was that the segmented data used in their study lacked physical interpretability. Despite such limitations, these studies show that fusing temporal and spatial features can significantly improve SOH estimation accuracy and provide a new direction for future performance optimization of battery management system.

Although impressive progress has been made in current research, most existing data-driven SOH estimation methods tend to ignore physical interpretability of feature sequence extraction and its spatial correlation. Therefore, there is a pressing need for a method that not only achieves high accuracy but also provides physical interpretability by leveraging both spatial and temporal degradation information. To address these issues, this paper proposes a novel method for SOH estimation in lithium-ion batteries, which combines a physically interpretable feature extraction technique based on IC analysis with a hybrid model that integrates GAT and bi-directional gated recurrent unit (BiGRU). The main contributions of this paper include:

- A novel physically interpretable feature extraction method is proposed. By dynamically selecting an optimal voltage window centered on IC peaks, the method ensures that the extracted feature fragments are directly linked to key electrochemical degradation mechanisms, enhancing the model’s interpretability.
- A hybrid GAT-BiGRU architecture is developed for synergistic spatial–temporal feature learning. To capture the complex relationships within the data, the framework first converts the time-series input into a graph structure and then GAT is employed to model the dependencies between these interconnected data segments while BiGRU captures their long-term temporal dependencies, offering a more comprehensive representation of the battery’s degradation processes.
- The proposed method’s superiority is rigorously validated on two public benchmark datasets, highlighting its strong generalization and robustness in handling diverse operational conditions. Furthermore, a detailed analysis of its computational footprint confirms its low operational complexity and rapid inference speed, demonstrating its suitability for deployment in resource-constrained embedded platforms.

The rest of the paper is organized as follows: Section 2 describes the methodology presented in this paper. Section 3 describes experimental setup. Section 4 describes experimental results and discusses them in detail. Section 5 summarizes work of this paper.

## 2. Methodology

**Figure 1:**
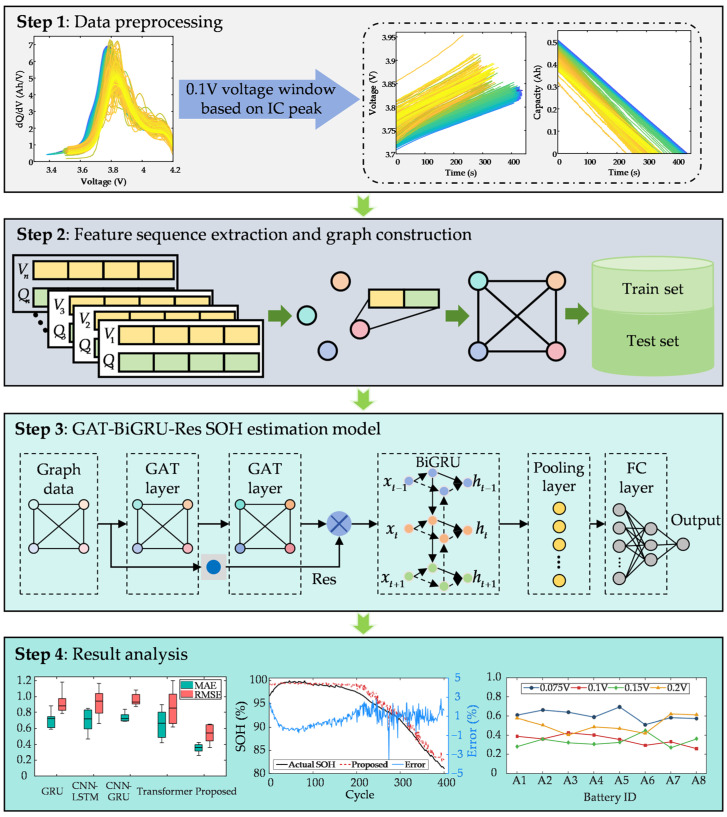
The framework of SOH estimation method.

To address the challenges of incomplete data acquisition during real-world charging operations and the limited interpretability in feature sequences, this paper proposes a lithium-ion battery SOH estimation method based on explainable feature segments using a GAT-BiGRU-Res model, with its framework illustrated in Figure 1. The specific process is as follows. Firstly, IC analysis is employed to dynamically track the temporal evolution patterns of IC curve peaks, adaptively determining a 0.1 V optimal voltage window for extracting voltage/capacity segment pairs. Next, interpolation aligns the voltage and capacity sequences, each of which is divided into four parts to capture local degradation features. Temporal data are then transformed into graph-structured data through graph modeling, with each sub-segment’s voltage–capacity feature vector serving as a graph node. Finally, the graph data are processed by the proposed model, where the GAT extracts spatial correlations among nodes, the BiGRU models temporal dependencies, and residual connections optimize gradient propagation. A fully connected layer outputs the discharge capacity estimate, thus enabling precise SOH estimation.

### 2.1. Data Preprocessing

The entire process of SOH estimation begins with the acquisition of operational data from the battery. Specifically, the voltage and current data used as the foundation for our analysis are captured in real-time by dedicated sensors integrated within the BMS during the charging process. Lin et al. [37] extracted feature sequences by selecting a fixed voltage range. However, this approach lacks a certain degree of flexibility and is prone to omit key aging information in view of the variability and complexity of the battery usage process. In addition, while various data-driven adaptive windowing techniques can identify statistically salient regions, they often fail to capture features with clear physical meaning, which hinders model interpretability. To overcome this limitation, this paper adopts a voltage segment from a dynamically selected voltage range based on the IC peak, along with the corresponding capacity data. This strategy significantly enhances the physical interpretability of the extracted features. Specifically, the peaks on an IC curve are direct manifestations of electrochemical phase transitions during lithium embedding and de-embedding, and their evolution is highly sensitive to degradation mechanisms. For example, reduced peak amplitudes correlate with decreased lithium-ion diffusion coefficients from cathode material cracking or electrolyte decomposition, while peak displacement reflects increased interfacial impedance due to solid electrolyte interphase film thickening at the anode. By dynamically centering the voltage window on the IC peak, this method ensures that the feature fragments are anchored to these physically meaningful events. This process guarantees that the same underlying aging phenomena are consistently captured, even as the peak’s position shifts due to degradation, thereby making the feature selection process transparent and scientifically grounded. The IC curve is defined as the rate of change in capacity with respect to voltage, and can be approximated by the following formula:

(1) $$IC(V_{k})=\frac{dQ_{k}}{dV_{k}}\approx \frac{Q_{k}-Q_{k-1}}{V_{k}-V_{k-1}}$$

where *k* is sampling interval, $Q_{k}$ and $V_{k}$ denote the capacity and voltage at the *k*-th sampling point.

In order to eliminate the effect of data noise, this paper adopts Gaussian filtering method, which has been proven to effectively smooth battery charging data [38]. The formula of Gaussian function is as follows:

(2) $$G(z)=\frac{1}{\sigma \sqrt{2\pi }}\exp\left(-\frac{{\left(z-\mu \right)}^{2}}{2\sigma^{2}}\right)$$

where z represents an individual data point from the input signal that is being smoothed, G(⋅) represents Gaussian function, which outputs the weight for a given input z, and μ and σ denote the mean and standard deviation of the data distribution, respectively. Then, the filtered value of each point is calculated by taking a weighted average of its neighboring points, using weights determined by the Gaussian function.

Then, the voltage fragment $V_{F}(t)$ and capacity fragment $Q_{F}(t)$ can be defined as:

(3) $$V_{F}(t)=[V_{\min},\;V_{\max}]$$

(4) $$Q_{F}\left(t\right)=\left[Q_{1},\;Q_{M}\right]$$

where $V_{\min}=V_{IC}-0.05$, $V_{\max}=V_{IC}+0.05$, $V_{IC}$ is the voltage at the IC peak position, $Q_{1}$ is the capacity at voltage $V_{\min}$, $Q_{M}$ is the capacity at voltage $V_{\max}$. Then, data standardization is carried out to eliminate effect of differences in data units.

### 2.2. Construction of Graph-Structured Data

To ensure consistency of the feature sequences across different cycles, cubic spline interpolation method is used to keep data aligned, and in this paper, the length of the feature sequences in each cycle is set to 80. In order to capture localized aging information in the feature sequence, feature fragments are further subdivided into four equal sub-segments for finer analysis of battery aging, which enhances the ability to characterize local changes and helps to gain insight into battery aging process. Each sub-segment represents a node in graph, then the node feature $y_{r}$ can be represented as:

(5) $$y_{r}=\left[V_{SF}(r)||Q_{SF}(r)\right],\;r=1,2,3,4$$

where || denotes vector concatenation, r represents the *r*-th node, $V_{SF}(r)$ and $Q_{SF}(r)$ denote the *r*-th sub-segment of voltage fragment and capacity fragment.

Then, the node identity matrix can be expressed as:

(6) $$Y=\left[y_{1};y_{2};y_{3};y_{4}\right].$$

The edges between these nodes are dynamically constructed based on the similarity of their feature vectors, for which cosine similarity is chosen. This selection is critical for capturing the essential attributes of battery degradation. The aging process often induces distinct morphological changes in the voltage and capacity profiles. Cosine similarity is adept at quantifying such morphological similarities by measuring the cosine of the angle between two vectors. This contrasts with Euclidean distance, which is sensitive to amplitude variations. Furthermore, while dynamic time warping (DTW) is a powerful tool for comparing sequences, its primary advantage lies in aligning series of different lengths. Our preprocessing step, which uses cubic spline interpolation to standardize segment lengths, obviates the need for DTW. Thus, cosine similarity provides a computationally efficient and physically meaningful measure of affinity between feature segments. The similarity matrices of voltage and capacity are first computed separately:

(7) $$A_{V}=\frac{V\cdot V^{T}}{{\left\|V\right\|}_{2}\times {\left\|V\right\|}_{2}}$$

(8) $$A_{Q}=\frac{Q\cdot Q^{T}}{{\left\|Q\right\|}_{2}\times {\left\|Q\right\|}_{2}}$$

where $V=[V_{SF}(1);V_{SF}(2);V_{SF}(3);V_{SF}(4)]$ and $Q=[Q_{SF}(1);Q_{SF}(2);Q_{SF}(3);Q_{SF}(4)]$, ${\left\|\cdot \right\|}_{2}$ denotes the 2-norm.

Subsequently, normalize the matrices and perform weighted fusion to generate the joint similarity matrix:

(9) $$A_{\mathrm{joint}}=\alpha \cdot \frac{A_{V}}{{\left\|A_{V}\right\|}_{2}}+\left(1-\alpha \right)\cdot \frac{A_{Q}}{{\left\|A_{Q}\right\|}_{2}}$$

where α denotes the joint weight.

Finally, we extract Top-*K* edges while excluding self-loops to construct adjacency matrix $A_{mn}$:

(10) $$A_{mn}=\left\{\begin{array}{l} A_{\mathrm{joint}}[m,n],\;\mathrm{if}\;n\in N_{K}\left(m\right)\;\mathrm{and}\;m\neq n\\ 0,\;\;\;\;\mathrm{otherwise} \end{array}\right.$$

where $N_{K}\left(m\right)$ denotes the top K maximum values in the *m*-th row of $A_{\mathrm{joint}}$, K denotes predefined maximum degree, which is 3, enabling the construction of a fully connected graph.

### 2.3. SOH Estimation Model

GAT is an advanced graph neural network architecture evolved from graph convolutional networks. It dynamically computes attention coefficients between target nodes and their neighbors through learnable mechanisms, rather than using predefined connection weights. This enables GAT to adaptively capture correlations and relative importance between nodes and their neighbors, leading to more accurate information aggregation from neighboring nodes and generating enriched node representations. Unlike conventional convolutional neural network (CNN), which is constrained to Euclidean data and uses static kernels, GAT is more adept at processing graph-structured data with non-Euclidean relationships. Its attention mechanism can dynamically learn the complex dependencies between different feature fragments, providing a more flexible and powerful approach for capturing non-local correlations. During battery aging, sub-segments exhibit heterogeneous degradation rates with inherent correlations. Therefore, employing GAT to extract latent dependencies between voltage sequences and capacity sequences from graph-structured data enables precise SOH estimation.

First, the nodes consisting of voltage and capacity sequences are linearly transformed using learnable weight matrix W in graph attention layer:

(11) $$h_{i}^{\prime }=Wh_{i}$$

where $h_{i}$ and $h_{i}^{\prime }$ denote the feature vectors of node *i* before and after transformation, respectively.

To capture dynamic correlation between nodes, attention coefficients of target node and its neighboring nodes are calculated:

(12) $$e_{ij}=\mathrm{LeakyReLU}\left(p^{T}\left[h_{i}^{\prime }||h_{j}^{\prime }\right]\right)$$

where p denotes the learnable parameter vector.

In order to eliminate effect of node degree differences, a function is used to normalize the attention coefficients to obtain final attention weights:

(13) $$\beta_{ij}=\frac{\exp\left(e_{ij}\right)}{\sum_{d\in \eta_{i}}\exp\left(e_{id}\right)}$$

where $\eta_{i}$ denotes neighbor set of node i.

To enhance the model representation, multi-head attention mechanism is used to learn node relationships in parallel. Each attention head independently calculates different inter-node weights to generate differentiated node representations from multiple perspectives:

(14) $$h_{i}^{\prime \prime \left(q\right)}=\varpi \left(\sum_{j\in N_{i}}\beta_{ij}^{\left(q\right)}W^{\left(q\right)}h_{j}^{\prime }\right)$$

where q denotes index of an attention head in the multi-head attention mechanism, ϖ denotes a nonlinear activation function.

Then, node information in the whole graph structure is summarized by averaging outputs of multiple attention heads in an operation. Meanwhile, in order to prevent loss of information caused by the GAT layers, Res is employed. By creating a skip connection that allows the gradient to flow directly, Res effectively mitigates the gradient vanishing phenomenon, which in turn enhances the model’s ability to portray nonlinear degradation trajectory of battery, ultimately yielding the final spatial feature vector. Critically, this is achieved via a computationally inexpensive element-wise addition, representing a highly favorable trade-off for the significant improvement in training stability.

To comprehensively capture bi-directional temporal dependencies in the spatial feature vectors extracted by the GAT layers, this work employs a specifically designed BiGRU architecture for temporal modeling. This architecture is selected for its effective balance of performance and computational efficiency, as it offers a comparable ability to capture long-range dependencies as more complex recurrent structures but with fewer parameters. The framework explicitly couples forward and backward GRU to dynamically integrate historical states and future evolution trends. This capability is particularly critical for battery degradation modeling because there exists an inherent causal relationship between past performance and future deterioration patterns. The bi-directional architecture enables the hidden state at each time step to be informed by the context of the entire sequence, resulting in a more robust and context-aware representation of the battery’s condition. This architecture consists of a single BiGRU layer, which is adept at capturing complex temporal patterns without excessive computational overhead. The hidden size for the forward and backward GRU is set to 80. Among them, the forward and backward process hidden layer is calculated as:

(15) $${\vec{h}}_{t}=GRU(x_{t},{\vec{h}}_{t-1})$$

(16) $${\overset{\leftarrow }{h}}_{t}=GRU\left(x_{t},{\overset{\leftarrow }{h}}_{t-1}\right)$$

The output calculation formula is as follows:

(17) $$h_{t}=w_{t}{\vec{h}}_{t}+v_{t}{\overset{\leftarrow }{h}}_{t}+b_{t}$$

where $x_{t}$ is the input at time *t*, $w_{t}$ denotes the hidden layer weights of the forward process, $v_{t}$ represents the hidden layer weights of the backward process, and $b_{t}$ is the output bias. The feature vectors processed by BiGRU layer are fed into fully connected layers, which transforms the hidden states into estimated discharge capacity.

## 3. Experimental Setup

### 3.1. Dataset

In this paper, the Xi’an Jiaotong University (XJTU) dataset [39] and Center for Advanced Life Cycle Engineering (CALCE) dataset [40] are selected for validation. The XJTU dataset consists of six sets of battery data, which were aged under different charging and discharging strategies. The batteries are 18,650 lithium nickel cobalt manganese-acid batteries and are manufactured by LISHEN (Tianjin, China), with a chemical composition of LiNi_0.5_Co_0.2_Mn_0.3_O_2_. They have a rated capacity of 2 Ah, a nominal voltage of 3.6 V, a charging cutoff voltage and a discharging cutoff voltage of 4.2 V and 2.5 V, respectively. The entire experiment was conducted at room temperature. In this paper, the first two groups of batteries are selected for analysis, i.e., Batch1 and Batch2, which contain 8 and 15 batteries, respectively. The CALCE dataset was released by the University of Maryland and we analyzed four prismatic LiCoO_2_ batteries from the CX2 series. The rated capacity is 1.35 Ah. They were aged at room temperature with constant current and constant voltage charging at 0.5 C and discharging at 1 C, with a sampling rate of 1/30 Hz.

During battery aging, the current degree of performance degradation of batteries is usually measured using capacity or internal resistance and expressed as a percentage [41]. Among them, capacity-based SOH is defined as:

(18) $$SOH=\frac{C_{aged}}{C_{0}}\times 100\%$$

where $C_{aged}$ and $C_{0}$ denote current maximum available capacity and rated capacity of battery, respectively.

**Figure 2:**
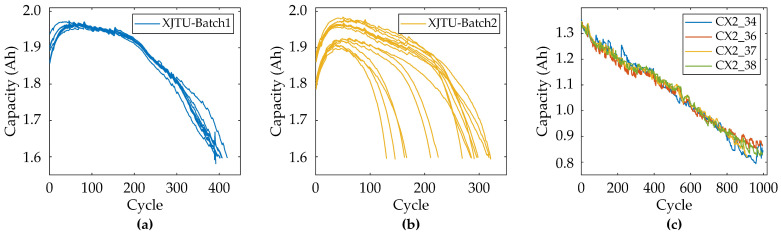
Capacity degradation trend. (**a**) XJTU-Batch1, (**b**) XJTU-Batch2, (**c**) CALCE-CX2.

**Figure 3:**
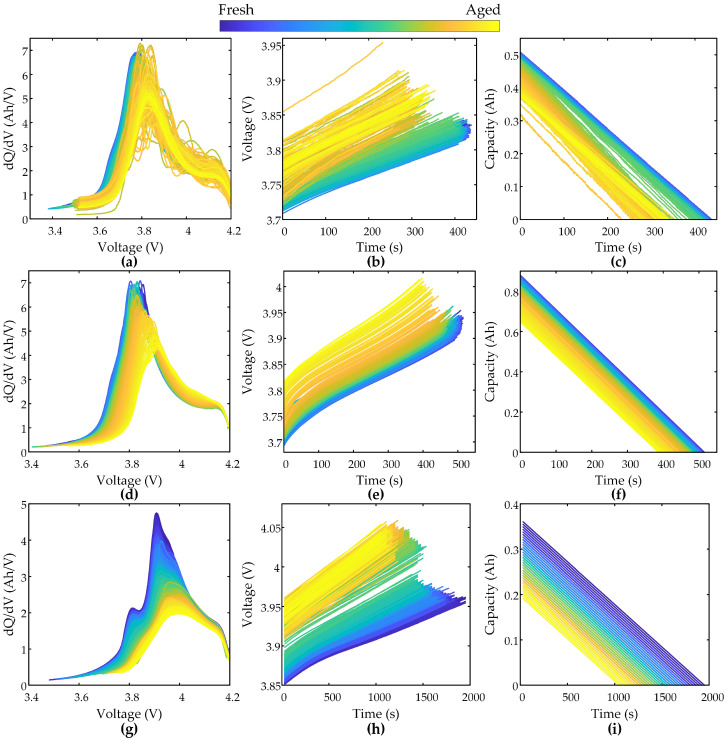
Evolution characteristics of feature fragments. (**a**–**c**) show IC curves, voltage segments, and capacity segments for XJTU-Batch1, with corresponding profiles for XJTU-Batch2 and CALCE-CX2 batteries shown in (**d**–**f**) and (**g**–**i**), respectively.

Figure 2 demonstrates the discharge capacity degradation curves for three battery datasets. As the cycle number increases, the discharge capacity of XJTU batteries decays gradually with accelerated degradation in late cycles, whereas CALCE batteries exhibit capacity regeneration phenomena during aging. Figure 3 illustrates the evolutionary characteristics of feature sequences across three battery datasets based on the data preprocessing method described in Section 2.1. Figure 3a,d,g display IC curves showing progressive southeastward shifts in IC peaks with aging. This rightward peak displacement reflects increased interfacial impedance due to solid electrolyte interphase film thickening at the anode, whereas reduced peak amplitudes correlate with decreased lithium-ion diffusion coefficients from cathode material cracking or electrolyte decomposition [42,43]. Figure 3b,e,h present dynamically extracted 0.1 V voltage windows centered on IC peaks, revealing rising initial voltages and shortened charging durations during aging, significantly indicating an increased charge voltage hysteresis induced by cathode phase transitions [2]. Figure 3c,f,i depict capacity sequences with diminished charging times and capacity increments, demonstrating irreversible lithium inventory loss from lithium plating or dead lithium formation and active material loss [44]. This mechanistic analysis confirms the selected feature segments effectively characterize battery degradation.

### 3.2. Model Parameter Settings

**Figure 4:**
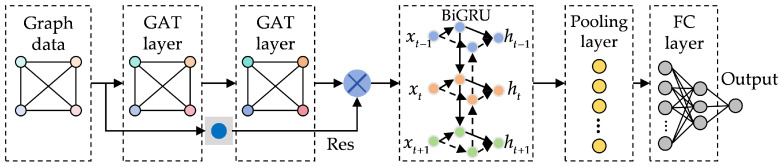
The overall structure of the proposed model.

Figure 4 and Table 1 illustrate structure and parameters of the proposed model, including two GAT layers, one BiGRU layer, one Res module and three fully connected (FC) layers. During training, Adam optimizer is used with an initial learning rate of 0.001, the learning rate is managed using a step learning rate scheduler, which reduces it by a factor of 0.5 every 10 epochs, and the number of iterations is set to 100. And loss function is a hybrid loss function of mean square error (MSE) and mean absolute error (MAE).

### 3.3. Evaluation Metrics

In order to quantitatively evaluate effectiveness of the proposed method, three evaluation metrics, MAE, root mean square error (RMSE) and determination coefficient (R^2^), are used. The calculation formulas are as follows:

(19) $$\mathrm{MAE}=\frac{1}{N}\sum_{i=1}^{N}\left|{\hat{C}}_{i}-C_{i}\right|$$

(20) $$\mathrm{RMSE}=\sqrt{\frac{1}{N}\sum_{i=1}^{N}{\left({\hat{C}}_{i}-C_{i}\right)}^{2}}$$

(21) $$R^{2}=1-\frac{\sum_{i=1}^{N}{\left({\hat{C}}_{i}-C_{i}\right)}^{2}}{\sum_{i=1}^{N}{\left({\bar{C}}_{i}-C_{i}\right)}^{2}}$$

where N denotes the number of cycles, $C_{i}$ and ${\hat{C}}_{i}$ represent the actual and estimated value of the SOH at *i*-th cycle, respectively, and ${\bar{C}}_{i}$ denotes the average SOH value across all cycles.

## 4. Results and Discussion

### 4.1. Results of the Proposed Method

**Figure 5:**
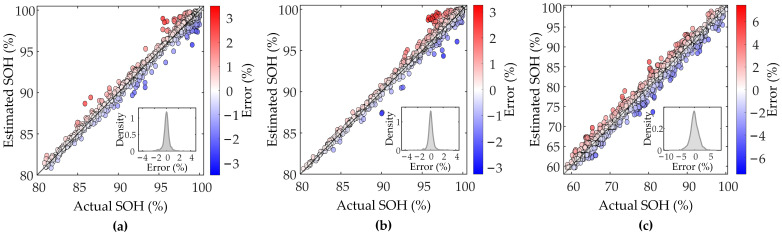
SOH estimation results of the proposed method. (**a**–**c**) present SOH estimation results for XJTU-Batch1, XJTU-Batch2 and CALCE-CX2, respectively.

To validate effectiveness of the proposed method, each battery’s data in the dataset is randomly divided into training and test sets in ratio of 6:4 to ensure generalization ability of the model under different data distributions. By reserving a substantial 40% of the data for the test set, we ensure a more robust assessment of the model’s performance on unseen data. The experiments are implemented using Python 3.9 on a device powered by a 12th-generation Intel iCore i7−12700 CPU, 32 GB RAM, and a single GeForce RTX 3050 GPU. To provide insight into the model’s computational demand during the training stage, we measured the time required to process individual battery data. The average per epoch training time for a single battery is 1.7 s on the XJTU-Batch1 dataset, 1.1 s on XJTU-Batch2, and 4.5 s on CALCE-CX2. Figure 5 demonstrates SOH estimation results of the proposed method.

The proposed method shows excellent SOH estimation performance under different charging conditions and material scenarios, indicating that the selected voltage and capacity series can effectively reflect the evolution of the batteries’ degradation trajectories. In XJTU and CALCE datasets, the estimation curves of the proposed method consistently and closely match actual capacity degradation curves, showing excellent tracking accuracy and stability. Even in the early and mid-late cycles, the proposed method can still accurately capture nonlinear changes in aging rate of the batteries and complex local capacity regeneration phenomena in the CALCE dataset, with less error volatility. This verifies high accuracy and wide applicability of the proposed method in SOH estimation, both in simple degradation trends and complex operating conditions. Based on the error results, it can be seen that the error distributions are close to normal, with peaks all close to zero, indicating that most of the estimation errors are close to zero. Specifically, the error ranges are between −3% and 3% for Batch1, between −4% and 4% for Batch2, and between −6% and 6% for CX2, where most of the data points are centered around ±1%, ±1%, and ±4%, respectively. These results verify validity and reliability of the proposed method on different datasets to accurately estimate SOH.

Table 2 shows average of the results of the proposed method for evaluation metrics in the three datasets. The results verify that the proposed method has advantages of high accuracy and stability, and the estimation results are highly consistent with the actual capacity degradation trajectory. Compared with the estimation accuracies of Batch1 and Batch2, the accuracy of CX2 decreases, which may be attributed to the more obvious capacity regeneration phenomenon in the whole process of the batteries in this dataset, and the degradation process is more complicated, leading to a slight decrease in accuracy. However, the model still maintains a high level of estimation accuracy, verifying its effectiveness in complex degradation scenarios.

The strong performance documented in Figure 5 and Table 2 is a direct consequence of the physically informed feature engineering strategy. As established in Section 2.1, the feature fragments are dynamically extracted from a voltage window anchored to the IC peak. The IC peak serves as a reliable proxy for internal electrochemical phase transitions, and its evolutionary characteristics, such as the reduction in peak amplitude and shifts in peak position, are highly correlated with key degradation mechanisms. Therefore, the high accuracy of the model stems from its ability to process inputs that directly quantify the progression of these critical aging modes. This linkage between the model’s inputs and the battery’s underlying electrochemistry provides a clear mechanistic explanation for its robust quantitative performance.

**Figure 6:**
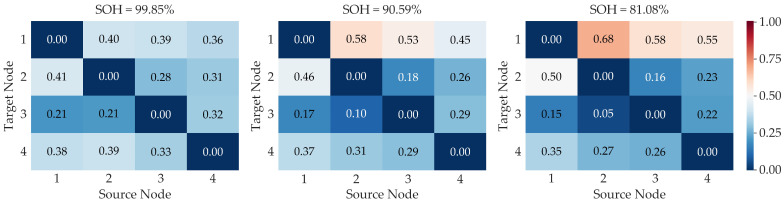
Evolution of GAT attention weights across different aging stages.

To provide insight into the model’s internal decision-making process, the attention mechanism of the GAT is visualized. Figure 6 displays the attention heatmaps for a representative battery at different aging stages selected based on SOH values. The results reveal a clear evolution in the model’s analytical priorities as the battery degrades.

In the early stage when aging characteristics are subtle, the model’s attention is relatively distributed across multiple feature relationships. As the battery enters the mid stage, a clear trend of prioritization emerges. The model assigns heightened importance to the information that target node 1 receives, particularly from source nodes 2 and 3. This indicates the model begins to identify the initial segment of the voltage window as a critical area for information integration. Finally, in the late stage, the analytical priority becomes highly specific. The attention that target node 1 pays to source node 2 becomes the single most dominant relationship in the matrix. This demonstrates that the model has successfully distilled the most critical indicator for severe degradation. This evolution from a broad analysis to a specific one showcases the model’s ability to dynamically learn and adapt its strategy, providing strong evidence for the interpretability of our proposed model.

### 4.2. Comparision of Methods

**Figure 7:**
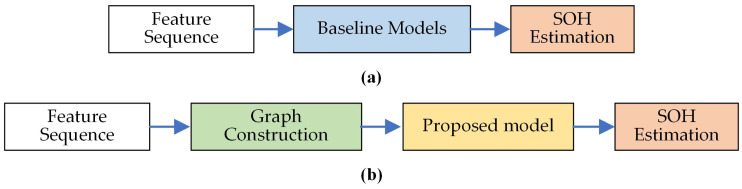
Schematic comparison of the architectures of the proposed method and baseline models. (**a**) presents the baseline models pipeline; (**b**) presents the proposed model pipeline.

**Figure 8:**
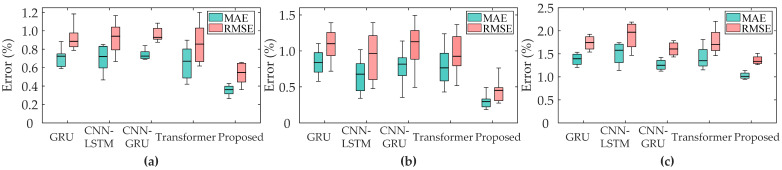
SOH estimation results of different methods. (**a**–**c**) present MAE and RMSE results for XJTU-Batch1, XJTU-Batch2 and CALCE-CX2, respectively.

In this paper, the benchmark deep learning methods GRU, CNN-LSTM, CNN-GRU and Transformer are utilized to compare with the proposed method, where the inputs of the comparison method are feature sequences created by concatenating the voltage and capacity sequences. The baseline models and the proposed method take fundamentally different architectural approaches to processing the input features. Figure 7 provides a schematic comparison of the data processing pipelines to visually illustrate these differences. The baseline models directly input the feature sequence into their respective architectures to learn temporal patterns. In contrast, the proposed method first transforms this sequence into a graph structure, enabling the explicit capture of spatial correlations between feature fragments before temporal modeling. The SOH estimation results are shown in Figure 8 and Table 3.

Figure 8 shows SOH estimation results of Batch1, Batch2 and CX2 in different methods, respectively. Table 3 presents the error metrics of different methods across datasets, with the best results for each dataset highlighted in bold. Our proposed method has the lowest MAE and RMSE, indicating its superior accuracy. As can be seen from the boxplots, the GRU method exhibits relatively high MAE and RMSE values, indicating substantial estimation errors. Although CNN-LSTM, CNN-GRU, and Transformer reduce average errors compared to GRU, they still underperform the proposed method. This suggests inherent limitations in conventional deep learning approaches for SOH estimation, particularly in effectively leveraging spatial feature information. Moreover, the proposed method maintains low errors across datasets, demonstrating strong stability and generalization. This indicates that the proposed method is highly adaptable to different types of battery data and can be applied to a variety of battery SOH estimation scenarios.

The preceding comparison demonstrates the superiority of the proposed method over conventional sequence-based models. However, a pertinent question arises as to whether this advantage stems primarily from the model’s architecture or from the graph-based data representation itself. To decouple these factors and facilitate a more direct comparison, a further experiment is conducted. In this experiment, the benchmark models are adapted to accept graph-structured inputs by integrating a GAT layer, thereby ensuring an identical input format across all models. The results of this augmented comparison are presented in Table 4.

A comparison between Table 3 and Table 4 reveals that the integration of a GAT layer and graph-structured inputs leads to a general improvement in the estimation accuracy of the benchmark models. For instance, the MAE of the GRU model on the XJTU-Batch1 dataset improved from 0.706% to 0.395%. This finding underscores the value of explicitly modeling the spatial correlations between feature fragments via the proposed graph construction strategy. Crucially, even under these modified conditions, the proposed GAT-BiGRU-Res model consistently exhibits the lowest error and highest R^2^ values across most datasets. This indicates that while the graph representation provides a significant benefit, the unique architectural combination of GAT for spatial feature extraction and BiGRU for capturing bi-directional temporal dependencies offers a distinct and synergistic advantage. The results suggest that this specific architecture is more effective at learning a comprehensive representation of the battery degradation process than simply applying a GAT layer to conventional sequence models.

### 4.3. Data Sensitivity Analysis

#### 4.3.1. Sensitivity to Voltage Ranges

**Figure 9:**
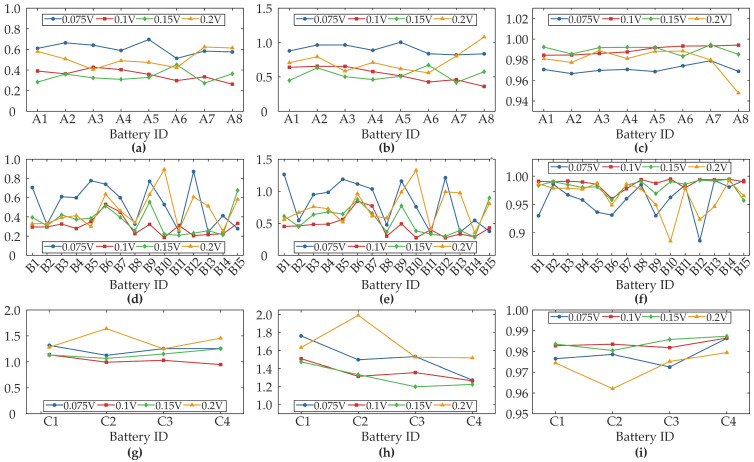
SOH estimation results of different voltage ranges. (**a**–**c**) present MAE, RMSE and R^2^ results for XJTU-Batch1, respectively; (**d**–**f**) present MAE, RMSE and R^2^ results for XJTU-Batch2, respectively; (**g**–**i**) present MAE, RMSE and R^2^ results for CALCE-CX2, respectively.

In order to investigate the effect of different voltage ranges on model performance, SOH estimation is validated for different voltage ranges. Specifically, four voltage window sizes were selected for comparative analysis: 0.075 V, 0.1 V, 0.15 V, and 0.2 V. Figure 9 demonstrates the impact of voltage window selection on SOH estimation performance for the proposed method. Table 5 presents the error results for different voltage ranges across datasets, where the best result for each dataset within each voltage range is highlighted in bold.

The results show that the 0.1 V range achieves optimal balance between accuracy and computational efficiency, capturing critical polarization features while avoiding noise sensitivity in shorter segments (0.075 V) and feature redundancy in longer segments (0.2 V). Although a 0.15 V range delivers comparable accuracy to the 0.1 V benchmark, the optimal choice is determined by factors related to practical implementation and robustness, as neither setting demonstrates a consistent performance advantage across all datasets. The primary differentiating factor is the data requirement. The 0.15 V window is substantially wider, which demands a larger, uninterrupted segment of charging data. This increased requirement can limit the algorithm’s applicability in real-world scenarios where charging sessions are often fragmented. Thus, employing the 0.1 V voltage window enables accurate SOH estimation across complex degradation scenarios, simultaneously ensuring model efficiency and enhancing deployment feasibility for practical battery management systems.

#### 4.3.2. Sensitivity to Graph Connectivity

To empirically validate the principled selection of K as justified in Section 2.2, we performed a sensitivity analysis by varying K from 1 to 3. This experiment aims to confirm that modeling the complete relational structure of the feature segments leads to optimal performance. The results are presented in Table 6.

The results in Table 6 provide strong empirical support for our selection. It demonstrates a monotonic improvement in model performance across all metrics as K increases from 1 to 3. This trend confirms that a fully connected graph enables the GAT to capture the most comprehensive set of spatial dependencies, thereby achieving the highest estimation accuracy. It also confirms the importance of graph density for the model’s ability to capture the intricate relationships between feature fragments. When K=1, each node aggregates information from only its single most similar neighbor, resulting in limited information flow and potentially preventing the model from perceiving global dependencies. As K increases, the graph becomes denser, allowing the GAT to aggregate features from a wider neighborhood when updating each node’s representation. Finally, at K=3, the graph is fully connected, enabling each node to consider the influence of all other nodes, thus forming the most comprehensive and holistic representation of the battery’s internal state. Therefore, a fully connected graph empowers the GAT to capture the most complete set of spatial dependencies, which yields the highest estimation accuracy.

#### 4.3.3. Sensitivity to the Number of Nodes

The number of feature sub-segments, which corresponds to the number of nodes in the constructed graph, is a critical hyperparameter that defines the model’s analytical granularity. This study proposes a 4-node configuration, a choice that represents a deliberate trade-off between model resolution and complexity. A coarser segmentation (e.g., 2 nodes) may oversimplify the representation of the battery’s degradation process and fail to capture important localized features. Conversely, an overly fine segmentation (e.g., 8 or 16 nodes) can increase model complexity and introduce data redundancy, potentially amplifying the effect of noise without yielding proportional gains in accuracy. To empirically validate the selection of four nodes, a sensitivity analysis is conducted to compare its performance against configurations with coarser and finer granularities. The results of this analysis are summarized in Table 7.

The results presented in Table 7 provide strong validation for the proposed 4-node configuration. For the XJTU_Batch1 and CALCE_CX2 datasets, the 4-node model achieves optimal performance, demonstrating the lowest MAE and RMSE values. While the 8-node configuration shows a marginally lower MAE for the XJTU_Batch2 dataset, the proposed 4-node model exhibits a superior RMSE and R^2^ value, indicating a more balanced and robust performance overall.

A consistent pattern emerges across all datasets: increasing the number of nodes from 2 to 4 yields a significant improvement in estimation accuracy. However, further increasing the granularity to 8 or 16 nodes provides no consistent or substantial benefits and, in several instances, leads to a minor degradation in performance. This trend suggests that the 4-node configuration effectively captures the essential characteristics of the aging process. Finer segmentations appear to offer diminishing returns, introducing complexity without enhancing the model’s ability to generalize. Therefore, this analysis confirms that the choice of four nodes is a well-justified selection, establishing an effective balance between feature resolution and model complexity.

### 4.4. Model Generalization Performance Analysis

**Figure 10:**
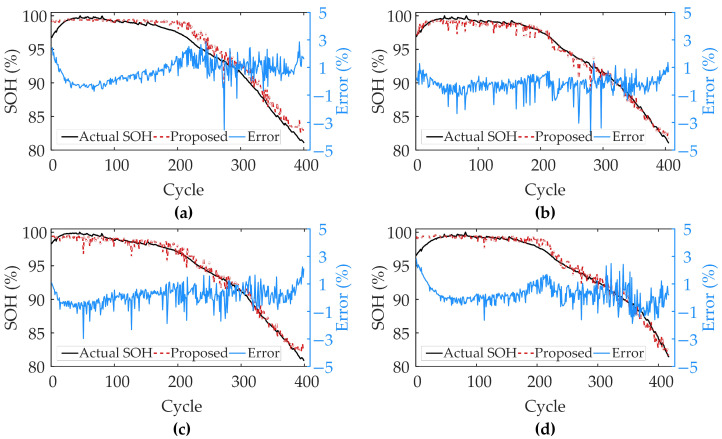
Cross-batteries SOH estimation results. (**a**–**d**) present SOH estimation results for batteries ID A5 to A8, respectively.

The generalization of the proposed method is further verified in this section by training the model on data from the first four batteries in Batch1 and testing it on the remaining batteries from the same batch. According to Figure 10, the proposed model performs well in tracking the dynamic changes in health state and is highly consistent with trend of the actual values, which verifies effectiveness of the proposed model in capturing the batteries degradation trend. The error analysis shows that the model maintains a high estimation accuracy during most of the cycles, despite large prediction bias in some cycles. In addition, consistency of the model’s performance on different batteries proves its good generalization ability to adapt to differences between different battery individuals. Table 8 shows that the proposed model provides reliable SOH estimation results on different batteries. The average MAE, RMSE, and R^2^ values for all batteries are 0.608, 0.802, and 0.977, respectively, which indicates that the proposed model achieves high accuracy across different batteries and effectively handles battery-to-battery variations, demonstrating good generalization capability.

### 4.5. Analysis of Model Deployment Feasibility

The practical utility of an SOH estimation algorithm is contingent not only on its accuracy but also on its feasibility for deployment on resource-constrained hardware. Therefore, we conducted an analysis of the proposed model’s deployment readiness, and metrics including network parameters, size, floating point operations (FLOPs), multiply–accumulate operations (MACs), inference memory, and inference time are evaluated, with the results summarized in Table 9. The results show that model’s architecture is relatively lightweight, comprising approximately 0.45 million parameters, requiring 1.68 MFLOPs per inference, and exhibiting a memory footprint increase of only 0.46 MB during execution. To test its real-world performance on an edge device, the model is executed on a Raspberry Pi 4 B (Raspberry Pi Ltd., Cambridge, UK), with a 1.5 GHz 64-bit quad-core Arm Cortex-A72 CPU and 8 GB of RAM. The test results show an average inference time of just 17.55 ms. In addition, the methodology utilizes charging data are standard outputs from the sensors universally equipped in commercial BMS hardware. The model’s requirement for low-frequency sampling is well within the typical operational parameters of these sensors. This confirms that the input data requirements do not pose a significant implementation challenge.

Collectively, these metrics provide strong evidence for the model’s deployment feasibility. The modest parameter count, low operational complexity, and fast inference speed on a representative embedded system strongly support its suitability for real-time SOH estimation on modern BMS platforms. This performance profile suggests the model is well-suited for contemporary hardware environments, while its application in systems with more severe resource constraints may warrant specific consideration.

### 4.6. Performance Evaluation with Noisy and Incomplete Data

To evaluate the model’s suitability for real-world BMS applications, where data can be imperfect, two experiments are conducted to assess the model’s robustness against sensor noise and data loss. The input feature sequences of the test set are modified for this purpose, and the results are summarized in Table 10 and Table 11.

First, to simulate the effect of sensor noise, additive white gaussian noise is added to the input features. The intensity of the noise is quantified using the signal-to-noise ratio (SNR) in decibels (dB). Three levels are tested: a low-noise condition (30 dB), a medium-noise condition (25 dB), and a high-noise condition (20 dB). As shown in Table 10, the model’s performance degrades gracefully as the noise level increases, demonstrating its resilience to measurement noise. To simulate intermittent data loss, a portion of the data points are randomly removed from the input sequences and subsequently filled using interpolation. The results in Table 11 show a similar trend of graceful degradation. The estimation error increases with the rate of missing data, but the model avoids catastrophic failure, underscoring its ability to extract stable features from incomplete data. This analysis confirms the model’s robustness, a critical attribute for practical deployment. However, the results also underscore that more reliable input data are crucial for optimal model performance. While the model demonstrates resilience by avoiding catastrophic failure, its estimation precision noticeably decreases under severe noise and high data loss rates. This reveals a practical limitation: the model’s reduced accuracy in these extreme scenarios might be insufficient for applications that require high-precision performance.

## 5. Conclusions

In this paper, a novel GAT-BiGRU-Res model for SOH estimation of Li-ion batteries based on feature fragment data are proposed and validated. The study successfully demonstrates an interpretable feature engineering approach where an aging-sensitive 0.1 V voltage window, dynamically selected via IC peak analysis, effectively extracts physically meaningful feature fragments. The proposed synergistic architecture, leveraging a graph attention network for spatial correlations and a bi-directional gated recurrent unit for temporal dependencies, is then shown to adeptly leverage these high-quality inputs. This integrated methodology achieves excellent accuracy with MAEs of 0.355%, 0.303%, and 1.025% on the XJTU-Batch1, XJTU-Batch2, and CALCE-CX2 datasets, respectively. From an industrial perspective, the significance of this work lies in its excellent balance between high accuracy and computational efficiency. With only 0.45 million parameters and requiring just 1.68 MFLOPs per inference, the model achieves a rapid average inference time of 17.55 ms on a representative embedded device. This quantified efficiency confirms its strong potential for deployment in real-time, resource-constrained embedded platforms, thereby enhancing the safety and reliability of energy storage systems.

While the current findings are promising, acknowledging the study’s scope is crucial for charting future research pathways. The model’s efficacy is established using widely recognized public benchmark datasets with standardized testing protocols. However, these protocols do not encompass all dynamic scenarios in real-world applications. Future work should therefore focus on three key areas: (1) expanding validation to a wider range of datasets that incorporate diverse operational scenarios, (2) investigating the influence of temperature on the degradation mechanisms reflected in the IC curve to develop temperature-adaptive models, and (3) exploring transfer learning techniques to facilitate rapid adaptation to new battery chemistries. By addressing these aspects, the proposed framework can be evolved into a more universally applicable and trustworthy solution for advanced battery diagnostics.

## Figures and Tables

**Table 1:** Parameters of the proposed model.

Module	Parameters
GAT1	in_channels = 40, out_channels = 160, heads = 4;
GAT2	in_channels = 640, out_channels = 320, heads = 1;
Res	in_features = 40, out_features = 320;
BiGRU	input_size = 320, hidden_size = 80;
Dropout	dropout = 0.2;
Pooling	hidden_size = 160;
FC1	in_features = 160, out_features = 64;
FC2	in_features = 64, out_features = 32;
FC3	in_features = 32, out_features = 1.

**Table 2:** Errors in the proposed method.

	XJTU_Batch1	XJTU_Batch2	CALCE_CX2	Average
MAE_ave (%)	0.355	0.303	1.025	0.561
RMSE_ave (%)	0.536	0.452	1.362	0.783
R^2^_ave	0.989	0.988	0.984	0.987

**Table 3:** Errors of the proposed method and other methods.

Dataset	Methods	MAE_Ave (%)	RMSE_Ave (%)	R^2^_Ave
XJTU_Batch1	GRU	0.706	0.918	0.967
	CNN-LSTM	0.702	0.922	0.969
	CNN-GRU	0.740	0.961	0.967
	Transformer	0.655	0.865	0.972
	Proposed	**0.355**	**0.536**	**0.989**
XJTU_Batch2	GRU	0.833	1.075	0.932
	CNN-LSTM	0.665	0.915	0.950
	CNN-GRU	0.805	1.058	0.934
	Transformer	0.772	0.959	0.946
	Proposed	**0.303**	**0.452**	**0.988**
CALCE_CX2	GRU	1.381	1.738	0.973
	CNN-LSTM	1.509	1.899	0.968
	CNN-GRU	1.262	1.606	0.977
	Transformer	1.415	1.768	0.972
	Proposed	**1.025**	**1.362**	**0.984**

Note: The best results are highlighted in bold.

**Table 4:** Performance comparison of the proposed model and GAT-enhanced benchmark models.

Dataset	Methods	MAE_Ave (%)	RMSE_Ave (%)	R^2^_Ave
XJTU_Batch1	GAT-GRU	0.395	0.611	0.986
	GAT-CNN-LSTM	0.581	0.867	0.969
	GAT-CNN-GRU	0.498	0.799	0.973
	GAT-Transformer	0.503	0.742	0.978
XJTU_Batch2	GAT-GRU	0.356	0.535	0.982
	GAT-CNN-LSTM	0.501	0.798	0.960
	GAT-CNN-GRU	0.875	1.216	0.917
	GAT-Transformer	0.544	0.774	0.962
CALCE_CX2	GAT-GRU	1.068	1.439	0.983
	GAT-CNN-LSTM	1.052	1.385	0.983
	GAT-CNN-GRU	1.083	1.423	0.983
	GAT-Transformer	1.040	1.383	0.983

**Table 5:** Errors of different voltage ranges.

Dataset	Voltage (V)	MAE_Ave (%)	RMSE_Ave (%)	R^2^_Ave
XJTU_Batch1	0.075	0.609	0.899	0.971
	0.1	0.355	0.536	0.989
	0.15	**0.337**	**0.527**	**0.990**
	0.2	0.515	0.732	0.979
XJTU_Batch2	0.075	0.536	0.821	0.959
	0.1	**0.303**	**0.452**	**0.988**
	0.15	0.361	0.551	0.982
	0.2	0.463	0.749	0.964
CALCE_CX2	0.075	1.238	1.516	0.979
	0.1	**1.025**	1.362	**0.984**
	0.15	1.150	**1.308**	**0.984**
	0.2	1.407	1.667	0.973

Note: The best results are highlighted in bold.

**Table 6:** Performance comparison for different *K*-values in graph construction.

Dataset	*K*	MAE_Ave (%)	RMSE_Ave (%)	R^2^_Ave
XJTU_Batch1	1	0.511	0.761	0.978
	2	0.471	0.721	0.980
	3	**0.355**	**0.536**	**0.989**
XJTU_Batch2	1	0.372	0.577	0.980
	2	0.366	0.571	0.981
	3	**0.303**	**0.452**	**0.988**
CALCE_CX2	1	1.062	1.414	0.982
	2	1.048	1.391	0.983
	3	**1.025**	**1.362**	**0.984**

Note: The best results are highlighted in bold.

**Table 7:** Performance comparison for different numbers of nodes.

Dataset	Number of Nodes	MAE_Ave (%)	RMSE_Ave (%)	R^2^_Ave
XJTU_Batch1	2	0.557	0.844	0.974
	4	**0.355**	**0.536**	**0.989**
	8	0.389	0.596	0.987
	16	0.398	0.618	0.986
XJTU_Batch2	2	0.307	0.457	0.987
	4	0.303	**0.452**	**0.988**
	8	**0.295**	0.455	0.987
	16	0.313	0.485	0.986
CALCE_CX2	2	1.015	1.380	0.983
	4	**1.025**	**1.362**	**0.984**
	8	1.037	1.380	0.983
	16	1.091	1.426	0.982

Note: The best results are highlighted in bold.

**Table 8:** Cross-batteries SOH estimation results errors.

	A5	A6	A7	A8	Average
MAE (%)	0.872	0.456	0.522	0.582	0.608
RMSE (%)	1.091	0.657	0.673	0.788	0.802
R^2^	0.962	0.985	0.985	0.974	0.977

**Table 9:** Deployment performance metrics of the proposed model.

Parameters	Size	FLOPs	MACs	Memory	Time
453057	1.76 MB	1.68 M	0.84 M	0.46 MB	17.55 ms

**Table 10:** Performance under various levels of additive white gaussian noise.

Dataset	SNR (dB)	MAE_Ave (%)	RMSE_Ave (%)	R^2^_Ave
XJTU_Batch1	30	0.690	1.070	0.950
	25	0.812	1.263	0.932
	20	1.425	1.884	0.857
XJTU_Batch2	30	0.786	1.005	0.937
	25	0.858	1.090	0.912
	20	1.130	1.478	0.860
CALCE_CX2	30	2.124	2.608	0.932
	25	2.319	2.873	0.922
	20	3.040	3.803	0.876

**Table 11:** Performance with different rates of missing data.

Dataset	Missing Data Rate (%)	MAE_Ave (%)	RMSE_Ave (%)	R^2^_Ave
XJTU_Batch1	5	0.866	1.192	0.942
	10	1.010	1.546	0.907
	15	1.291	1.972	0.854
XJTU_Batch2	5	0.643	1.106	0.932
	10	0.929	1.226	0.905
	15	1.243	1.668	0.837
CALCE_CX2	5	1.937	2.550	0.910
	10	2.221	2.748	0.890
	15	2.279	2.765	0.859

## Data Availability

These data were derived from the following resources available in the public domain: the XJTU dataset available at https://wang-fujin.github.io (accessed on 15 April 2025), reference number [39] and the CALCE dataset available at https://calce.umd.edu/data (accessed on 15 April 2025), reference number [40].

## Abbreviations

The following abbreviations are used in this manuscript:

SOH	State of health
IC	Incremental capacity
GAT	Graph attention network
BiGRU	Bi-directional gated recurrent unit
Res	Residual connection
EVs	Electric vehicles
BMS	Battery management system
LSTM	Long short-term memory network
GRU	Gated recurrent unit
GNN	Graph neural network
DTW	Dynamic time warping
XJTU	Xi’an Jiaotong university
CNN	Convolutional neural network
CALCE	Center for advanced life cycle engineering
FC	Fully connected
MSE	Mean square error
MAE	Mean absolute error
RMSE	Root mean square error
R^2^	Determination coefficient
FLOPs	Floating point operations
MACs	Multiply–accumulate operations
SNR	Signal-to-noise ratio
k	Sampling interval
$Q_{k}$	Capacity at the *k*-th sampling point
$V_{k}$	Voltage at the *k*-th sampling point
$IC(V_{k})$	IC value at $V_{k}$
z	Individual data point from the input signal that is being smoothed
G(⋅)	Gaussian function
μ	Mean of the filtered data distribution
σ	Standard deviation of the filtered data distribution
$V_{F}\left(t\right)$	Voltage fragment
$Q_{F}\left(t\right)$	Capacity fragment
$V_{IC}$	Voltage at the IC peak position
$V_{\min}$	Starting point of voltage fragment
$V_{\max}$	Ending point of voltage fragment
$Q_{1}$	Starting point of capacity fragment
$Q_{M}$	Ending point of capacity fragment
$y_{r}$	Node feature
$V_{SF}(r)$	*r*-th sub-segment of voltage fragment
$Q_{SF}(r)$	*r*-th sub-segment of capacity fragment
Y	Node identity matrix
$A_{V}$	Similarity matrices of voltage
$A_{Q}$	Similarity matrices of capacity
$A_{\mathrm{joint}}$	Joint similarity matrix
α	Joint weight
$A_{mn}$	Adjacency matrix
K	Predefined maximum degree of adjacency matrix
W	Learnable weight matrix of GAT
$h_{i}$	Feature vectors of the node before GAT transformation
$h_{i}^{\prime }$	Feature vectors of the node after GAT transformation
$e_{ij}$	Attention coefficients of target node and its neighboring nodes
p	Learnable parameter vector of GAT
$\beta_{ij}$	Final attention weights
$\eta_{i}$	Neighbor set of node *i*
$h_{i}^{\prime \prime }$	Final node representation
q	Index of attention head in multi-head attention mechanism
$x_{t}$	Input to BiGRU
$w_{t}$	Hidden layer weights of BiGRU forward process
$v_{t}$	Hidden layer weights of BiGRU backward process
$b_{t}$	Output bias of BiGRU
$h_{t}$	Feature vectors processed by BiGRU layer
$C_{aged}$	Current maximum available capacity
$C_{0}$	Rated capacity
N	Total number of charging cycles
$C_{i}$	Actual value of the SOH at *i*-th cycle
${\hat{C}}_{i}$	Estimated value of the SOH at *i*-th cycle
${\bar{C}}_{i}$	Average SOH value across all cycles
